# A Novel and Safe Approach to Simulate Cutting Movements Using Ground Reaction Forces

**DOI:** 10.3390/s18082631

**Published:** 2018-08-11

**Authors:** Amelia S. Lanier, Brian A. Knarr, Nicholas Stergiou, Thomas S. Buchanan

**Affiliations:** 1 Biomechanics and Movement Science Program, College of Engineering, University of Delaware, Newark, DE 19716, USA; alanier@unomaha.edu (A.S.L.); bknarr@unomaha.edu (B.A.K.); 2 Department of Biomechanics and Center for Research in Human Movement Variability, College of Education, University of Nebraska at Omaha, Omaha, NE 68182, USA; nstergiou@unomaha.edu; 3 Delaware Rehabilitation Institute, University of Delaware, Newark, DE 19716, USA; 4 Department of Environmental, Agricultural & Occupational Health, College of Public Health, University of Nebraska Medical Center, Omaha, NE 68198, USA

**Keywords:** biomechanics, movement control, anterior cruciate ligament, kinetics, real-time feedback

## Abstract

Control of shear ground reaction forces (sGRF) is important in performing running and cutting tasks as poor sGRF control has implications for those with knee injuries, such as anterior cruciate ligament (ACL) ruptures. The goal of this study was to develop a novel and safe task to evaluate control or accurate modulation of shear ground reaction forces related to those generated during cutting. Our approach utilized a force control task using real-time visual feedback of a subject’s force production and evaluated control capabilities through accuracy and divergence measurements. Ten healthy recreational athletes completed the force control task while force control via accuracy measures and divergence calculations was investigated. Participants were able to accurately control sGRF in multiple directions based on error measurements. Forces generated during the task were equal to or greater than those measured during a number of functional activities. We found no significant difference in the divergence of the force profiles using the Lyapunov Exponent of the sGRF trajectories. Participants using our approach produced high accuracy and low divergence force profiles and functional force magnitudes. Moving forward, we will utilize this task in at-risk populations who are unable to complete a cutting maneuver in early stages of rehabilitation, such as ACL deficient and newly reconstructed individuals, allowing insight into force control not obtainable otherwise.

## 1. Introduction

In athletic populations, the knee is a commonly injured region of the body, with younger athletes being especially at risk [1,2]. Overall, strain or sprain injuries are extremely prevalent and occur at a rate of 102 incidents per 100,000 athletes per year [3]. These types of injuries typically occur when athletes are cutting or landing from a jump [4,5]. Additionally, ankle injury models estimated that one-third of ankle injuries occur during a sharp turn or twist [6]. Medial-lateral force generation during a sharp turn or twist is additionally noted to be greater in those with functional instability of the ankle joint, emphasizing the importance of accurate shear ground reaction force (sGRF) modulation [7]. Cutting and turning tasks require modulation of sGRFs. To turn during gait, an individual first decelerates to generate a posteriorly directed GRF, and then adjusts the GRF medially or laterally to change direction [8]. In more dynamic tasks like cutting, Havens and colleagues found significant correlations between medial-lateral GRF impulse and cutting angle and performance [9,10] while Jones and colleagues found that medial/lateral GRFs significantly correlated to peak knee adduction moment during cutting and pivoting tasks [11]. sGRFs significantly dictate gait and cutting maneuvers [12,13,14], and so the ability to control these forces are especially important when considering their implications on other joints as the forces propagate through the body. For example, traumatic knee injuries such as anterior cruciate ligament (ACL) ruptures are often a result of simultaneous multidirectional loading involving sGRFs [15]. 

Many studies that aim to understand injury recovery utilize biomechanical measures (joint angle, joint translations, etc.) in conjunction with evaluation of their variability using analyses such as variance [16,17,18,19]. These analyses investigate changes among many individual features within a trial, and may focus on the amount of variation that occurs around a central point (i.e., mean and standard deviation) or singular features of the cycle (i.e., maximum or minimum). These analyses do not capture the differences in evolution of the signal over time that may occur from cycle to cycle during continuous movements. However, these subtle time varying changes that are identified through the analysis of many continuous cycles may be crucial to understand recovery, as they can indicate neuromuscular health [20]. To capture these subtle changes, a different approach can be utilized, where tools such as the Lyapunov Exponent (LyE) [21,22,23,24] can describe the temporal structure of various time series data. Larger values of the LyE indicate more divergence in the trajectories of the continuous movement cycles, while smaller values indicate less divergence. The LyE has been successfully used in numerous biomechanical applications [22,23,25]. Research focused on ACL injury and recovery indicates greater divergence (larger LyE) of knee flexion angle movement trajectories during continuous gait cycles in ACL deficient and reconstructed limbs when compared to the intact limb [21,26]. Greater kinematic divergence is present in ACL reconstructed limbs in all reconstruction types, indicating less control of the joint [21]. Alterations to divergence of knee flexion angle movement trajectories during gait post ACL injury and reconstruction may indicate functional deficits exist. However, such an evaluation was never performed with kinetic data to provide a more comprehensive picture of the changes that may occur following such an injury. 

This study aims to develop and evaluate a novel and safe approach to allow participants to generate and control shear ground reaction forces similar to those generated during cutting. This study is the first but necessary step to demonstrate feasibility for a larger study that aims to understand force control via accuracy and divergence in injured populations. This study is important as it establishes the first dataset of normative/baseline force control in a healthy, uninjured population, which will allow us to better understand changes in force control as a result of injury. To understand force control of shear ground reaction forces we have developed a task using real-time visual feedback of a participant’s force production. For this task to be deemed valuable, participants must be able to accurately control sGRFs (as defined by current literature), must be able to perform this task in multiple directions, must generate meaningful/functional force magnitudes, and must exhibit similar divergence of trajectories across directions and limbs. To first address force control measured via accuracy, we analyzed mean absolute error in both limbs and all directions. Based on studies utilizing a force matching task of different joints [27,28,29], we defined an allowable amount of absolute error to be below 10% maximum effort (MAX). To then address task performance in multiple directions, we assessed force magnitudes in both limbs and all directions. We hypothesized that task completion, as evaluated by both force magnitude and absolute error, would be similar between limbs. To determine if the force control task produced functional loads, target force magnitudes relative to subject mass were also calculated. We hypothesized that force magnitudes generated during the force control task would be greater than or equivalent to those produced during functional activities. Lastly, we wanted to be able to evaluate force control capabilities of the participants by investigating divergence of the data. Variability was analyzed by calculating the standard deviation of the absolute error of forces generated during the task for each limb and all directions. We hypothesize that the standard deviation of the absolute error will be similar across both limbs and directions during the force control task. Force control measured via divergence was also analyzed by calculating the LyE using force profiles generated during the task. We hypothesize that there will be no difference in force control measured via divergence (using the LyE) when comparing the LyE of the right and left limb or directions of movement (anterior/posterior and medial/lateral).

## 2. Materials and Methods

### 2.1. Participants

Ten healthy individuals with no previous history of knee injuries were included: three men (age 22 ± 0.8 years, BMI 22.9 ± 0.7 kg/m^2^) and seven women (age 22 ± 0.3 years, BMI 21.9 ± 2.3 kg/m^2^). One subject was removed from the nonlinear analyses (*n* = 9), as they did not follow instructions to generate a smooth force pattern. This participant generated non-continuous (extreme asymptotic) force profile, which significantly impedes our ability to reliably and accurately quantify the Lyapunov Exponents. The subject was used for linear calculations (*n* = 10), as they still attempted to successfully produce the maximum forces displayed by the visual feedback. All participants were active in >50 h/yr of level I and II sports, which include running and cutting activities. This study was approved by the institutional review board at the University of Delaware and all participants provided written informed consent. 

### 2.2. Force Control Task

**Figure 1:**
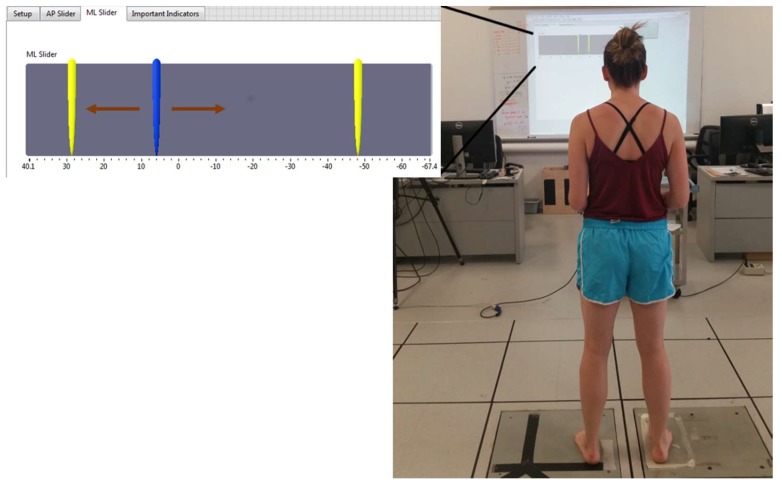
Experimental setup of data collection (**right**): example of visual feedback provided to study participants during the medial/lateral (ML) force control task (**left**): arrows indicate the direction the mobile cursor moves.

All participants completed a force control task. For the force control task, participants stood on two separate force platforms (AMTI OR-6, Watertown, MA, USA) with a foot on each force plate. It should be noted that the foot morphology of each participant was not recorded, which may have affected foot placement. Throughout testing and calibration trials, the participants were in regular stance (Figure 1) facing the screen with feet positioned approximately hip width distance apart. Participants received real-time visual feedback of their force production in the leg of interest via custom written Labview code. Visual feedback of the participant’s force production was presented on a screen in front of them. To visualize force production, a slider was utilized. A slider is an object that moves in response to a signal. The visual feedback format included a slider that responded to force production and two stationary indicators that identified the target force for the participants (Figure 1). The two stationary indicators represented 50% of the participant’s maximum strength in that direction, based on the maximal push trials (Figure 1). 

To calibrate the force control task to each participant’s strength, the participants performed maximal force production trials. All participants completed the ‘maximal push’ trials in all shear directions (anterior, posterior, medial, and lateral) with each foot, separately. Participants were instructed to push as hard as they were able to in the four directions described above. All participants completed the ‘maximal push’ trials in all shear directions with each foot. After completing the ‘maximal push’ trials, the feedback software was calibrated, and participants began the force control testing. 

Participants were instructed to control the slider and align it with the two stationary indicators. They were instructed to generate force bi-directionally (anterior/posterior or medial/lateral) and continuously to the beat of a metronome set at 60 beats per minute, and to alternatively align the slider with each stationary indicator. Participants were instructed to align the slider to each indicator alternatively and continuously and not to simply surpass the indicators. Additionally, participants were instructed to continue a cyclical and smooth trajectory if there were unable to reach the indicator. Participants were told to move to the next indicator if unable to reach the other. The force control task was two minutes in duration. Conducting the trial at 60 beats per minute for two minutes in duration was chosen to generate enough data to calculate the LyE while minimizing the duration of the trial. The force control task was conducted for both the right and left limbs and both the anterior/posterior and medial/lateral directions. This lead to a total of four conditions for each leg. Subjects were only restricted in their foot placement but were allowed to position the other joints (hip, knee, ankle) freely throughout all testing and calibration. Subjects were instructed to maintain their foot placement in the same location throughout testing. To maintain foot placement, we traced or placed tape in specific locations on the plate so subjects were able to move between trials and still maintain the same foot placement across all trials. 

### 2.3. Data Analysis

To evaluate force control during this task, we evaluated both accuracy and divergence of the force profiles. To evaluate accuracy, we calculated a number of error measures. Using the calibration of maximal push trials and force profiles measured during the force control task, we calculated the absolute error (% Max). Absolute error was measured using custom Matlab software as the absolute value of the difference between the peak and target force for each trial. Using the maximal push trial, we recorded the maximum force production in each direction, which were then used to create the threshold levels for the indicators. From the force profiles collected during the force control trials, we compared the force generation at each peak to the 50% Max threshold. Any overshooting or undershooting of the target force was considered as an error. We calculated both the mean and standard deviation of the absolute error for both limbs (right and left) and all directions (anterior, posterior, medial, and lateral). Confidence intervals of 95% were also calculated for target force, absolute error, standard deviation of absolute error, and the LyE. In addition to our error measurements we evaluated force magnitudes per kg of body mass (N/kg) in the four directions (anterior, posterior, medial, and lateral) that were tested.

**Figure 2:**
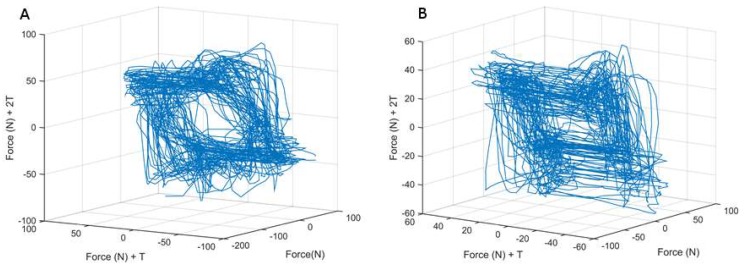
Depiction of trajectories of continuous movement cycles of larger and smaller Lyapunov Exponents. (**a**) Movement trajectories with a smaller Lyapunov Exponent; (**b**) Movement trajectories with a larger Lyapunov Exponent.

The Lyapunov exponent (LyE) was calculated using the force profiles produced during both the anterior/posterior (AP) or medial/lateral (ML) force control tasks in a manner similar to previous research [21,22,23,26] (Figure 2) to determine AP and ML force divergence, respectively. In all cases, unfiltered data was used to get a more accurate representation of the data, as filtering may remove subtle changes within the signal [30,31] (Figure 2). Data analysis was performed at 60 Hz. Spectral analysis revealed that the signal of interest primarily existed at 6 Hz and lower, and data was sampled at 10× this frequency range (60 Hz) to ensure that sufficient points were used in the analysis without oversampling the data. We also note that a 60 Hz sampling rate is commonly used when analyzing human movement [32]. The LyE is defined as the rate of divergence of infinitesimally close trajectories [33], and is determined through a multi-step process. 

**Figure 3:**
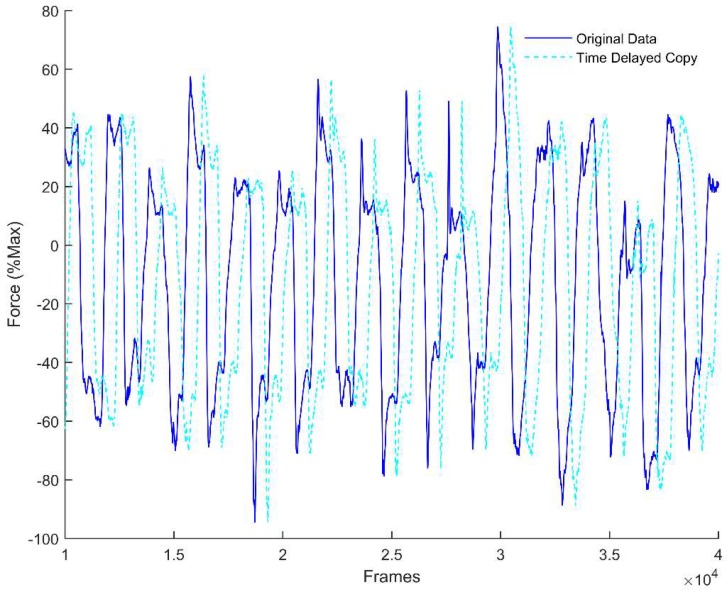
Original force profile (solid line) with a time delayed copy (dashed line).

**Figure 4:**
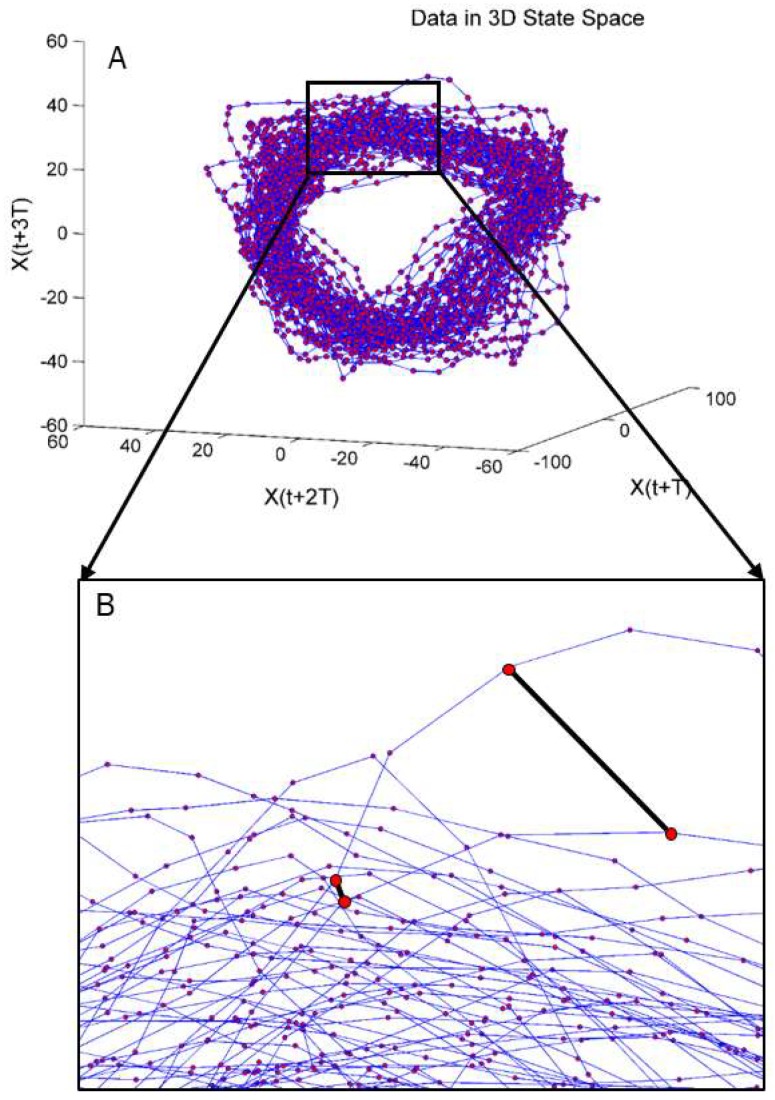
Force profile data X(t) transformed into 3D state space (**A**). Zoomed in view (**B**) with the distance between trajectories highlighted in black. X(t+T) represents force data shifted by the time lag, tau.

Calculating the LyE requires two input parameters: time lag (*τ*) and embedding dimension (*m*). Both of these parameters were used to convert our signals of interest into state space. To calculate *τ*, we used the Average Mutual Information (AMI) function which determines the percentage of information shared between two signals [34]. For this study, the two signals used in the AMI function were the force profile (either in the AP or ML direction) and a copy of the same force profile (Figure 3). To determine *m*, the Global False Nearest Neighbor (GFNN) function was used [34]. Using time delayed copies (as specified by *τ*) of a signal, GFNN measures the distance between trajectories in one dimension, and then increases the number of dimensions and measures again (Figure 4). A false nearest neighbor is defined by any significant changes in distance between trajectories when increasing the number of dimensions. As the dimensions are increased, the number of false neighbors is determined. The first local minima of the GFNN function was then used as *m*. GFNN is important to ‘unfold’ the data, as any folding would lead to misinterpreted LyE. Once *τ* and *m* are determined, the data are transformed in state space; in state space, the LyE can then be calculated. For this data set, each force profile was the same number of data points (3600), *m* was constant across trials and conditions at a value of five, while *τ* was calculated for each trial (*τ* = 18 *±* 0.35). The LyE was calculated using the Wolf et al., algorithm, as this algorithm is more sensitive to changes in variability when using small data sets [32,33]. This algorithm calculates the Euclidean distance between trajectories and tracks the trajectories forward in time to determine the rate of divergence or convergence of the trajectories. It is important to note that trajectories when using LyE analysis refers to the evolution of the analyzed signal over time, which in this study is force production over time, not position data, which is sometimes used. The rate of divergence is determined for multiple trajectories and the largest, which is our value of interest, is reported. The LyE values were calculated for each subject for both the AP and ML direction providing two measures, AP LyE and ML LyE. 

### 2.4. Statistical Analysis

To evaluate force control via accuracy, multiple statistical tests were performed. Descriptive statistics including minimum, maximum, and average values were also calculated for absolute error and target force. Two separate two-by-four repeated measures (limbs: right vs. left by direction: anterior vs. posterior vs. medial vs. lateral) ANOVA were conducted to determine significant differences between the group means of the target force and absolute error. For both target force and absolute error, specific pairwise comparisons within the ANOVA framework were evaluated to control for type I error inflation at a significance level of 0.05. Additionally, an upper level *t*-test was conducted to determine if the mean absolute error was greater than the allowable 10% MAX as determined by previous literature [21,22,23]. Lastly, a post-hoc power analysis was conducted for the absolute error values.

To further evaluate force control during the force control task via accuracy, a two-by-four repeated measures (limbs: right vs. left by direction: anterior vs. posterior vs. medial vs. lateral) ANOVA was conducted to determine significant differences for the standard deviation of the absolute error of forces generated. Lastly, a two-by-two (limbs: right vs. left by direction: AP vs. ML) fully repeated measures ANOVA was used to determine significant differences between the group means for the LyE and the standard deviation of the absolute error. One subject was removed from the LyE analysis as they were unable to generate a smooth force trajectory as they were instructed to do so. Post-hoc analysis was performed for any tests that resulted in significant interaction. The significance level was set at 0.05 and analysis was performed using SPSS (IBM Armonk, NY, USA).

## 3. Results

In our evaluation of both absolute error of forces generated and target force, all participants completed the force control task similarly across limbs (Table 1 and Table 2). There was no significant effect of limb based on repeated measures ANOVA when comparing the means of both target force (*p* = 0.117) and absolute error (*p* = 0.813). Our analysis of target force revealed a significant effect of direction (*p* < 0.001). Target force ranged from an average of 38.53 N (left posterior) to 64.29 N (left medial). Our analysis of absolute error indicated no significant effect of either direction or interaction. 

**Figure 5:**
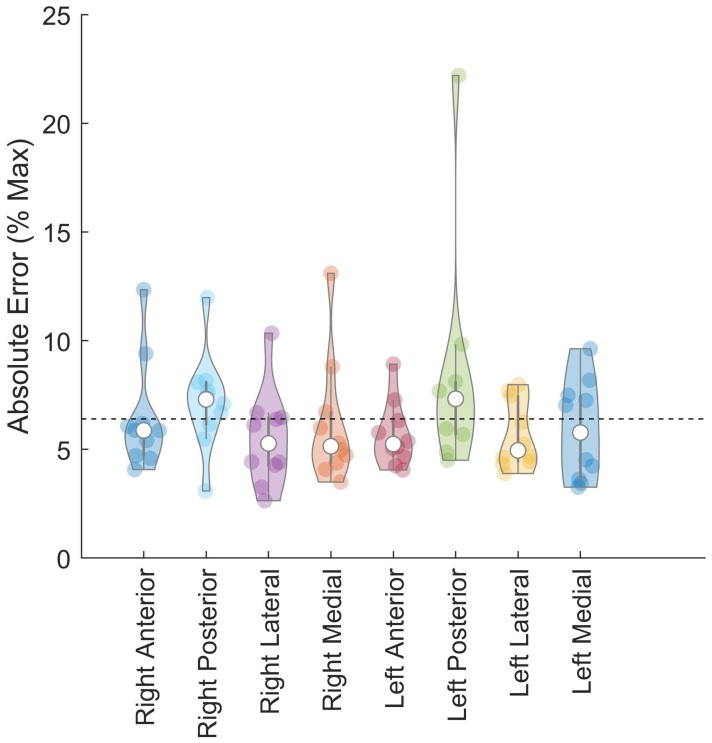
Violin plot of absolute error (% Max) during the force control task for each limb (right, left) and all directions (anterior, posterior, medial, lateral). Median absolute error indicated by open circle, interquartile ranges are represented by thick and thin vertical lines, and overall average is indicated by dashed line. Shaded circles represent individual subject data. * *p* < 0.05 (one-tailed *t*-test).

Confidence intervals of 95% and average absolute error of forces generated during the force control task were below the 10% MAX allowable error (Table 1 and Table 2, Figure 5). *t*-test results indicate that the average absolute error was below the 10% target (*p* < 0.001). The minimum average absolute error calculated was 5.5% MAX from the right limb in the lateral direction, while the maximum average absolute error generated was 8.35% MAX in the left limb in the posterior direction (Table 1). For each limb and direction, the average absolute error did not exceed the 10% MAX threshold, however absolute error of forces generated from the left limb in the posterior direction did exhibit large standard deviations which did exceed the 10% MAX threshold when considering standard deviations in absolute error. The post-hoc power analysis indicated sufficient power for seven out of the eight measures (seven variables (1 − β) ≥ 0.98, average Cohen’s d = 1.96; Absolute Error Left Limb Posterior Direction (1 − β) = 0.24, Cohen’s d = 0.32).

During the force control task, participants generated loads greater than that of dynamic functional tasks (Table 1). During the force control task, participants generated 0.54 N/kg, 0.60 N/kg, 0.77 N/kg, and 0.89 N/kg in the anterior, posterior, medial, and lateral directions, respectively (Table 1). Current literature reports force magnitudes ranging from 0.1–0.7 N/kg from a spectrum of activities including walking, running, and jumping [35,36].

**Figure 6:**
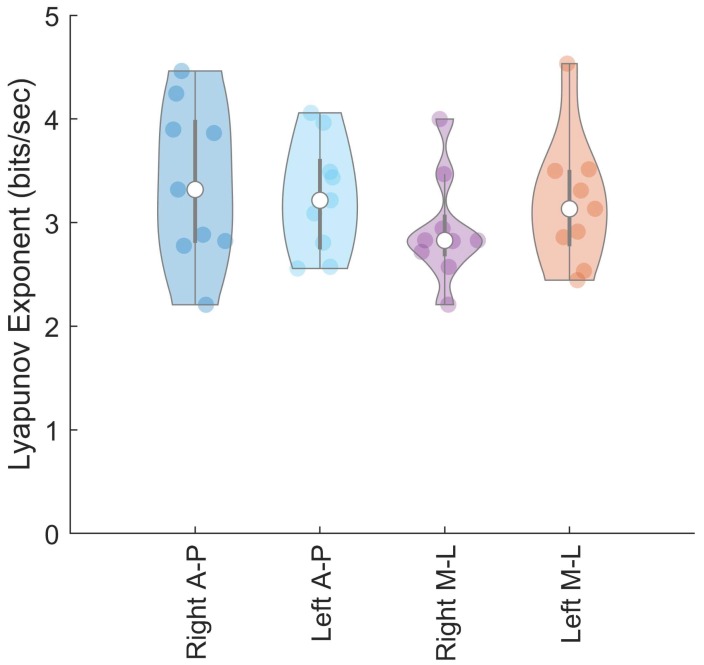
Violin plot of maximal Lyapunov exponent (bits/second) during the force control task. Median Lyapunov exponent indicated by open circle, interquartile ranges are represented by thick and thin vertical lines Shaded circles represent individual subject data. Data is reported for both the medial/lateral (M-L) and anterior/posterior (A-P) directions.

Measures of variability indicate no differences in force control capabilities between limbs (Table 1, Figure 6). Repeated measures ANOVA comparing the standard deviation of forces generated revealed no significant effect of limb (*p* = 0.783), direction (*p* = 0.132), or interaction (*p* = 0.498, Table 1). 

Based on the LyE calculated during the force control task, force control capabilities were similar across limbs for all participants (Figure 6). Our data indicate that there is no statistically significant difference in LyE values of healthy uninjured participants when comparing the right and left limb. In the right limb of healthy uninjured participants, we calculated the LyE values to be 3.39 ± 0.77 bits/s and 2.93 ± 0.52 bits/s in the AP and ML directions, respectively (Figure 6). In the left limb, we calculated the LyE to be 3.24 ± 0.55 bits/s and 3.19 ± 0.63 bits/s in the AP and ML directions (Figure 6). Additionally, our data indicate there is no statistically significant difference in the LyE values generated in the ML versus AP direction. *p*-values from the ANOVA approached significance (*p* = 0.054), but effect size calculated using Cohen’s d was moderate. 

## 4. Discussion

The aim of this study was to develop and evaluate a novel and safe approach to allow participants to generate and control shear ground reaction forces relatable to those generated during cutting. To meet this aim, we developed the force control task and evaluated it using a young, healthy and active cohort. Using real-time visual feedback of a participant’s force production, we aimed to establish a task where participants are able to control ground reaction forces in multiple directions at magnitudes similar to activities of daily living. To evaluate the force control task, we measured force accuracy via absolute errors. To evaluate functional relevance, we evaluated target force and force production relative to body mass. To evaluate force control, we explored variability using standard deviation of absolute error and we explored divergence using the Lyapunov Exponent. 

Our results indicate that participants were able to accurately control the multidirectional force production during the force control task. On average participants were accurate within 10% MAX with both limbs in all directions tested. Absolute error was consistent across the anterior, medial, and lateral directions with values ranging from 5.50% MAX to 6.45% MAX. Based on absolute error calculations, participants were least accurate in the posterior direction, particularly when generating force with the left limb (8.35% MAX absolute error). Larger errors generated by the left limb in the posterior direction maybe a result of strength deficits in that direction as noted by the target force which was weakest in the posterior direction (Table 1). Overall error values were below 10% MAX, across limbs and directions, indicating that participants were able to both accurately control their sGRF production in multiple directions during the force control task. This is an important and necessary finding to demonstrate that the task is achievable and feasible in a healthy population and can provide a normative baseline for evaluating performance in injured and rehabilitating populations. 

A secondary goal of this task was for participants to generate forces relatable to running and cutting maneuvers. As we were unable to find literature investigating sGRF production of cutting, there are numerous studies utilizing other similar tasks which have been included. Results from this study and current research indicate that the force control task produces functionally relevant force magnitudes. Force profiles from this task reveal force generation at magnitudes larger than that of dynamic functional tasks. Participants generated forces ranging from 0.54–0.89 N/kg, with current literature reporting force magnitudes ranging from 0.1–0.7 N/kg for activities including walking, running, and jumping. AP GRF produced during gait initiation and running initiation ranges from 0.2–0.7 N/kg, while ML GRF produced gait termination ranges from 0.1–0.5 N/kg [35,36]. During a vertical jump landing, ML GRF ranges from 0.1–0.4 N/kg [35]. After analyzing calibration trials collected for this study, we found participants generate 0.54 N/kg (anterior), 0.60 N/kg (posterior), 0.77 N/kg (medial) and 0.89 N/kg (lateral) when completing the force control task. Additionally, studies utilizing a similar force control task estimated force magnitudes of ranging from 0.48 to 0.60 N/kg [36]. During the force control task, participants generate forces that are equal to or greater than that of functional tasks like walking and running. From these results, we determined that the force control task was able to be dynamically challenging and provide meaningful data on sGRF production in groups that perform high level dynamic tasks like cutting. 

In our calculation of the Lyapunov Exponents, we found no statistically significant difference in force control for divergence between limbs when performing the force control task. These results were to be expected as our participants endured no major injuries to their lower limbs. Additionally, we found no statistically significant differences in force control for trajectory divergence (LyE) between directions, but participants did exhibit slightly reduced LyE values when performing the force control task in the ML direction. This may indicate better control in that direction, as a lower LyE value indicates less divergence. However, data from additional populations are needed to understand the magnitude of these differences in force control regarding divergence. These results indicate that healthy uninjured participants are able to maintain proper force control in each limb and in different directions when force control is measured via trajectory divergence. 

There were some limitations in the implementation of this study. First, force control accuracy task was limited to 50% of each participant’s maximum force generation, and it could be assumed that at higher levels of force production, there would be a decrease in accuracy. However, to limit the potential for injury, a lower level of force production was desired. While no participants indicated that they were unable to see the visual feedback, use of contact lenses or glasses were not recorded. In future studies, potential visual impairment will be recorded. During the force control task, no fail criteria was established to ensure participants completed the task to a certain level of accuracy. However, during data collection, the variables of interest were being monitored, and in subsequent data analysis, all trials were inspected for both the magnitude of each subject’s force production and the appropriate number of cycles. By monitoring the number of cycles, we were able to ensure that the participants maintained appropriate timing. 

In this study, we developed a novel task where participants accurately control sGRFs similarly with the right and left limb at functionally meaningful magnitudes in a low risk and dynamic setting. Error measured during the task was within tolerance based on related research, and divergence measures calculated during the task revealed no significant differences between limbs. We have established a standard for force control as participants generate sGRFs that can be used as a basis for comparison in other demographic and pathological populations. It is important to establish a normative baseline when using Lyapunov Exponent analysis as interpretation of the data necessitates comparisons between groups to identify trends. While a number of studies have explored the changes to the temporal structure in kinematic measures, no work has been done utilizing kinetic measures. The design of this task allows for application in at-risk populations, such as ACL deficient and newly reconstructed individuals, as the task is quasi-static but still demanding in respect to forces generated. This is important as it allows insight into the production of forces similar to functional activities, such as cutting, that would be not be obtainable otherwise. Future work will seek to understand changes to force control that may occur as a result of ACL injury and reconstruction or participation in high performance athletics, with the ultimate goal of informing both rehabilitation practices and sports training. 

## Figures and Tables

**Table 1:** Error calculations (mean ± standard deviation) for both the right and left limb in all directions tested during the force control task for healthy uninjured participants.

	Right Anterior	Left Anterior	Right Posterior	Left Posterior	Right Medial	Left Medial	Right Lateral	Left Lateral
Target Force (N)	41.11 ± 10.63	43.79 ± 12.88	41.72 ± 11.35	38.53 ± 9.52	54.35 ± 13.22	64.29 ± 12.05	64.07 ± 10.67	55.56 ± 12.85
Abs. Error (% Max)	6.45 ± 2.53%	5.68 ± 1.49%	7.19 ± 2.27%	8.35 ± 5.12%	6.16 ± 2.87%	5.65 ± 1.55%	5.5 ± 2.21%	5.87 ± 2.30%
St. Dev. Abs. Error (% Max)	4.86 ± 1.70%	4.50 ± 1.17%	5.62 ± 1.96%	5.77 ± 2.73%	4.31 ± 1.24%	4.28 ± 1.11%	4.09 ± 1.40%	4.59 ± 1.52%
Force (N/kg)	0.54 ± 0.21		0.60 ± 0.23		0.77 ± 0.28		0.89 ± 0.31	

**Table 2:** Confidence intervals of 95% for all error calculations in all directions tested during the force control task for healthy uninjured participants.

	Anterior	Posterior	Medial	Lateral
Target Force (N)	37.03–47.87	35.29–44.96	49.01–60.90	58.99–69.36
Abs. Error (%)	5.10–7.03%	5.94–9.60%	4.82–7.20%	4.71–6.45%
St. Dev. Abs. Error (%)	4.01–5.35%	4.61–6.78%	3.81–5.08%	3.61–4.76%
LyE	2.99–3.64		2.78–3.35	
